# BayFish: Bayesian inference of transcription dynamics from population snapshots of single-molecule RNA FISH in single cells

**DOI:** 10.1186/s13059-017-1297-9

**Published:** 2017-09-04

**Authors:** Mariana Gómez-Schiavon, Liang-Fu Chen, Anne E. West, Nicolas E. Buchler

**Affiliations:** 1 Program in Computational Biology & Bioinformatics, Duke University, Durham, NC USA; 2 Present address: Department of Biochemistry & Biophysics, University of California, San Francisco, CA USA; 3 Department of Neurobiology, Duke University, Durham, NC USA; 4 Department of Biology, Duke University, Durham, NC USA; 5 Department of Physics, Duke University, Durham, NC USA; 6 Center for Genomic & Computational Biology, Duke University, Durham, NC USA

**Keywords:** Gene expression, Stochastic process, Chemical master equation, Likelihood methods, Monte Carlo sampling, Bayesian posterior probability

## Abstract

Single-molecule RNA fluorescence in situ hybridization (smFISH) provides unparalleled resolution in the measurement of the abundance and localization of nascent and mature RNA transcripts in fixed, single cells. We developed a computational pipeline (BayFish) to infer the kinetic parameters of gene expression from smFISH data at multiple time points after gene induction. Given an underlying model of gene expression, BayFish uses a Monte Carlo method to estimate the Bayesian posterior probability of the model parameters and quantify the parameter uncertainty given the observed smFISH data. We tested BayFish on synthetic data and smFISH measurements of the neuronal activity-inducible gene *Npas4* in primary neurons.

**Electronic supplementary material:**

The online version of this article (doi:10.1186/s13059-017-1297-9) contains supplementary material, which is available to authorized users.

## Background

Cell-to-cell variation in gene expression across an isogenic population is a fact of life. The initiation of transcription involves a series of stochastic biochemical events, which includes chromatin accessibility, the binding of transcription factors, and the assembly of RNA polymerase at the promoter of a gene [1]. Distinct promoter states can often arise when one of these biochemical events is rate-limiting. The existence of multiple promoter states with different expression rates can generate transcriptional bursting, which are episodes of transcriptional activity followed by long periods of inactivity [2–4]. This phenomenon has been observed in bacteria [5, 6], yeast [7, 8], flies [9, 10], and mammals [11–16].

Cell-to-cell variability in gene expression is often studied using techniques that measure transcription in single cells. One such technique, single-molecule RNA fluorescence in situ hybridization (smFISH) measures the abundance and localization of individual transcripts in a single cell [17, 18]. This method uses a cocktail of fluorescently labelled DNA oligos complementary to the target RNA and works in many organisms; see [19]. Each individual transcript is bound by fluorescent DNA probes and appears as a bright, diffraction-limited spot in a fluorescence microscope. When there are multiple transcripts (e.g., active transcription sites, TSs, at gene loci), the measured intensity can be significantly brighter. The smFISH technique is simple and has been rapidly adopted by other labs to address cell-to-cell variability in gene expression. This has been helped in part by software packages [20, 21] that facilitate image segmentation and spot analysis.

Gene expression is dynamic and the properties of transcriptional bursting must be inferred from smFISH data, which are static snapshots or distributions of mRNA and active TSs per cell sampled from a population. This inference is done using mathematical models of stochastic gene expression, whose predicted distributions of transcripts and active TSs in a population of cells are then fitted to smFISH data to infer the model parameters and likely properties of transcriptional bursting [22]. The simplest model that generates such bursting is the two-state model, which presumes that a gene stochastically switches between two promoter states, a transcriptionally active state and an inactive state. The advantage of the two-state model is that the distributions have been solved analytically and model parameters are inferred by fitting moments (e.g., mean and variance) or the full distributions to the observed smFISH data using least-squares approaches. Despite the success of two-state models, more complicated models are often needed to explain the observed distributions properly [7, 15, 16, 23]. These complex models often do not have analytical solutions and one must resort to simplifying assumptions or computationally intensive numerical methods to calculate distributions [24]. This is especially true for genes that are not in a steady state, e.g., induced genes.

**Fig. 1:**
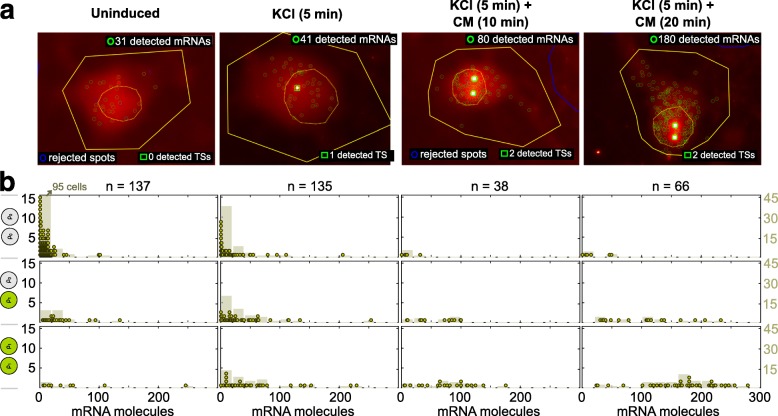
Single-molecule fluorescence in situ hybridization data of *Npas4* mRNA in primary neurons after membrane depolarization. Measurements are shown before the stimulus (uninduced), 5 min after KCl exposure, and an additional 10 and 20 min later after cells were returned to the conditioned medium (CM); see “Methods”. **a** Example of an image-processed cell at each time point. We show detected mRNAs (green circles) and active transcription sites (TSs; green squares) within the cell contour (yellow line) and nucleus (dashed yellow line). Neurons are post-mitotic and, thus, we observed up to two active gene loci per diploid cell. **b** For each condition, a histogram of the number of mRNA molecules binned by the number of TSs (dots, left *y*-axis). Smoothed histogram with bins of 20 mRNAs (bars, right *y*-axis). The total number of cells (*n*) per time sample is listed at the top. CM conditioned medium, TS transcription site

We used smFISH to measure transcripts of the neuronal activity-inducible gene *Npas4* in primary neurons after membrane depolarization with elevated extracellular potassium (Fig. 1). Our *Npas4* smFISH measurements showed a surprising amount of cell-to-cell variation in both transcript levels and active TSs even when all neurons were exposed to a uniform external stimulus. Given prior studies of cell-to-cell variability in gene expression in other systems, this variability in the transcriptional response of activity-inducible genes is likely to arise from the probabilistic activation of transcriptional bursting at single alleles. We reasoned that we could use this single-cell transcriptional variability to build a model of activity-inducible *Npas4* induction that would inform our quantitative understanding of the transcriptional processes that drive dynamic changes in *Npas4* expression following neuronal activation.

Thus, we developed a computational pipeline (BayFish) that uses a Bayesian approach to infer the best model parameters from smFISH data and to quantify the uncertainty in those parameters rigorously. The user specifies any mathematical model of stochastic gene expression with an unknown set of parameters (*θ*) and provides smFISH data (*Y*) at different time points before and after induction. BayFish then uses a Monte Carlo method to estimate the Bayesian posterior probability *P*(*θ*|*Y*) of the model parameters, which elucidates the best-fitting parameters and quantifies their uncertainty given the current smFISH data. We first tested BayFish on synthetic data and demonstrate how to select the best model from multiple mathematical models by combining information criteria with the likelihood and Bayesian posterior calculated by BayFish. We then used BayFish on the *Npas4* smFISH data to infer the parameters of an underlying two-state model of gene expression that were likely affected by the stimulus. Our results show that a two-state promoter model can recapitulate *Npas4* dynamics after induction and we further inferred that the transition rate from the promoter off state to the on state is increased by the stimulus.

There is currently no software that allows a user to specify any model of stochastic gene expression, evaluate the time evolution of mRNA and active TS distributions after induction, and rigorously infer parameters and confidence intervals from smFISH data using the Bayesian posterior probability. We expect BayFish to fill an important gap that will facilitate the adoption of the smFISH technique by other laboratories that wish to address cell-to-cell variability in gene expression.

## Results

BayFish is a software package that combines numerical methods with a Monte Carlo method to estimate the Bayesian posterior probability *P*(*θ*|*Y*) of model parameters (*θ*) given the observed smFISH data (*Y*) at different time points before and after induction. Bayes theorem states that *P*(*θ*|*Y*)=*P*(*Y*|*θ*)*P*(*θ*)/*P*(*Y*) where *P*(*Y*|*θ*) is the likelihood $\mathcal {L}$ of the data given the parameters. *P*(*θ*) and *P*(*Y*) are the prior probability distributions of the parameters and the data, respectively. Each iteration of the Monte Carlo method uses several numerical subroutines to calculate the time evolution of the mRNA and active TS distributions given a set of model parameters (*θ*), to evaluate the likelihood that the smFISH data (*Y*) were sampled from this distribution or ${\mathcal {L} = P(Y | \theta)}$, and to calculate the Bayesian posterior probability $\mathcal {P} = P(\theta | Y)$ given the likelihood and priors. The global program is based on the Metropolis random walk algorithm [25, 26]:

1. Specify a mathematical model of stochastic gene expression that has an unknown set of parameters *θ*.
2. Choose an initial *θ* and calculate the corresponding likelihood $\mathcal {L} = P(Y | \theta)$ and Bayesian posterior probability $\mathcal {P} = \mathcal {L} P(\theta) / P(Y)$ using several numerical subroutines.
3. Iterate over *t*={1,2,…,*T*} as follows:
  - Draw a random proposal $\phi \sim \theta _{t} + \mathcal {N}(0,\Sigma)$, where $\mathcal {N}(0,\Sigma)$ is a multivariate normal distribution with the same dimension as *θ* and with zero mean. *Σ* is the covariance matrix.
  - Evaluate the likelihood of the proposal $\mathcal {L}_{\phi } = P(Y | \phi)$ using several numerical subroutines.
  - Calculate the Bayesian posterior probability $\mathcal {P}_{\phi } = \mathcal {L}_{\phi } P(\phi) / P(Y)$.
  - Update parameters *θ*_*t*+1_←*ϕ* and $\mathcal {P}_{t+1} \leftarrow \mathcal {P}_{\phi }$ with probability $\text {min}(\mathcal {P}_{\phi } / \mathcal {P}_{t},1)$; otherwise, *θ*_*t*+1_←*θ*_*t*_ and $\mathcal {P}_{t+1} \leftarrow \mathcal {P}_{t}$.

Over time, the algorithm will generate a Markov chain of *θ*_*t*_ whose distribution converges to the Bayesian posterior probability *P*(*θ*|*Y*). BayFish saves the likelihood $\mathcal {L}_{t}$ and *θ*_*t*_ of each step. After discarding the early part of the chain (the burn-in phase), the remaining *θ*_*t*_ values were used to estimate the Bayesian posterior probability *P*(*θ*|*Y*); see “Methods.”

### Mathematical model of stochastic gene expression

**Fig. 2:**
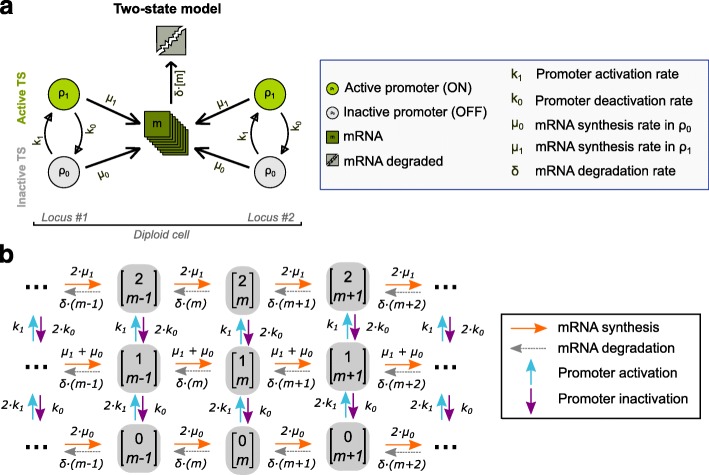
A two-state model of gene expression. **a** Each diploid cell has two genetic loci and the promoter (circle) of each gene can be in either an active (*ρ*_1_) or an inactive (*ρ*_0_) state. Each gene synthesizes mRNA molecules (*m*) with rate *μ*_1_ or *μ*_0_ if the promoter is active or inactive, respectively. Transitions between promoter states occur with a promoter activation rate *k*_1_ and a promoter deactivation rate *k*_0_. Each mRNA is degraded with rate *δ*. **b** Possible biochemical reactions and cell states of our model. A cell state **x** (grey box) is the number of active promoters *ρ*_1_∈{0,1,2} and mRNA molecules *m*∈{0,1,2,…,*M*} in a cell, or **x**=[*ρ*_1_,*m*]^*T*^. There are four possible biochemical reactions that change a cell from one state to another state: (1) promoter activation (blue arrow), which increases *ρ*_1_ by 1; (2) promoter inactivation (purple arrow), which decreases *ρ*_1_ by 1; (3) mRNA synthesis (orange arrow), which increases *m* by 1; and (4) mRNA degradation (gray arrow), which decreases *m* by 1. The propensity or probability per unit time (*a*_*k*_) for a particular reaction (*k*) to occur is listed above the reaction arrows. The propensities depend on the model parameters *θ*={*μ*_0_,*μ*_1_,*k*_1_,*k*_0_}

We considered a two-state model of gene expression (Fig. 2), where each promoter can be in an inactive off state *ρ*_0_ with a basal transcription level (synthesis rate *μ*_0_) or an active on state *ρ*_1_ with a higher transcription level (synthesis rate *μ*_1_). Transitions between promoter states occur with a promoter activation rate *k*_1_ and a promoter deactivation rate *k*_0_. We chose a two-state model because it is the simplest model that can generate transcriptional bursting, a feature observed in our *Npas4* smFISH data (Fig. 1). Neurons are post-mitotic and, thus, our model does not include duplicated alleles (e.g., three or four active loci) that arise after DNA replication. Each promoter allele was assumed to be regulated independently, as shown previously [11, 15, 23, 27]. The two-state model parameter set, which determines the dynamics of mRNA and active promoters, is *θ*={*μ*_0_,*μ*_1_,*k*_1_,*k*_0_}. We fixed the mRNA degradation rate *δ* because it is a known quantity, but this parameter could be a free parameter in other models.

Our smFISH experiments measured gene expression both before and after the stimulus. We presumed that gene expression before the stimulus was at a steady state determined by one set of model parameters (*θ* ^*U*^, unstimulated parameter set). Upon induction, the stimulus changed one or more of the model parameters (*θ* ^*S*^, stimulated parameter set). Thus, the mRNA and active TS distribution will evolve towards a new steady state in response to the changed parameters. Below, we describe how we calculated the stationary mRNA and active TS distribution before the stimulus using *θ* ^*U*^ and how we then calculated the time evolution of the distribution after the stimulus using *θ* ^*S*^.

### Time evolution of the probability distribution

The chemical master equation (CME) is an infinite set of coupled differential equations that describe the dynamics of the probability of the biochemical system being in a particular state ***x*** at time *t*, *P*(***x***,*t*) [28, 29]. The probability flow into and out of each state ***x*** is given by:

1 $$ \frac{\partial P(\boldsymbol{x},t)}{\partial t} = \sum\limits_{k} [a_{k}(\boldsymbol{x}-\boldsymbol{\nu_{k}}) P(\boldsymbol{x}-\boldsymbol{\nu_{k}},t) - a_{k}(\boldsymbol{x}) P(\boldsymbol{x},t)].  $$

The summation is over all possible biochemical reactions *k* into and out of state ***x***:

2 $$ \boldsymbol{x} \xrightarrow{a_{k}(\boldsymbol{x})} \boldsymbol{x} + \boldsymbol{\nu_{k}}  $$

where *a*_*k*_(***x***) *∂* *t* is the probability that the biochemical reaction *k* will occur within the infinitesimal time interval *∂* *t* given that the system is in state ***x***. The model parameters *θ* affect the propensities of different biochemical reactions (Fig. 2), and the stoichiometric vector (***ν***_***k***_) of reaction *k* describes how the system state changes when the reaction *k* occurs. More generally, the CME is written in matrix form:

3 $$ \frac{\partial \boldsymbol{P}(\boldsymbol{X},t)}{\partial t} = \boldsymbol{A}(\theta) \cdot \boldsymbol{P}(\boldsymbol{X},t)   $$

where all possible cell states ***X*** are enumerated as a vector [***x***_***1***_,***x***_***2***_,…,***x***_***N***_]^*T*^. ***P***(***X***,*t*) is the probability density state vector [*P*(***x***_***1***_,*t*),*P*(***x***_***2***_,*t*),…,*P*(***x***_***N***_,*t*)]^*T*^ of possible states organized identically to ***X***. The state reaction matrix ***A***(*θ*) has elements:

4 $$ \mathbf{A}_{ij} = \left\{ \begin{array}{ll} -{\sum\nolimits}_{k} a_{k}(\boldsymbol{x_{i}}), & \forall i = j, \\ a_{k}(\boldsymbol{x_{i}}), & \forall j \text{ such that}\,\, \boldsymbol{x_{j}} = \boldsymbol{x_{i}} + \boldsymbol{\nu_{k}}, \\ 0, & \text{otherwise.} \end{array} \right.  $$

#### Pre-stimulus stationary distribution

We assumed that the pre-stimulus mRNA and active TS distribution ***P*** ^∗^(***X***) is time-independent and stationary. We calculated the stationary distribution by setting Eq. 3 to zero and determined the nonzero eigenvector **V**≥**0** in the kernel of **A**(*θ* ^*U*^) using the Arnoldi iteration algorithm [30] (eigs MATLAB function, or eig_gen Armadillo C++ library). Each element of ***P*** ^∗^ is given by

5 $$ P^{*}(\boldsymbol{x}_{i}) = \frac{V_{i}}{{\sum\nolimits}_{j} V_{j}}  $$

where *V*_*i*_ is the *i*th element in the vector ***V***=[ *V*_1_,*V*_2_,…,*V*_*N*_]^*T*^ and ${\sum \nolimits }_{i} P^{*}(\boldsymbol {x_{i}})=1$.

#### Post-stimulus distribution dynamics

Given an initial distribution ***P*** ^∗^(***X***) at time zero and post-stimulus state reaction matrix ***A***(*θ* ^*S*^), the post-stimulus distribution ***P***(***X***,*τ*) at time *τ* after stimulus is:

6 $$ \boldsymbol{P}(\boldsymbol{X},\tau) = \operatorname{exp}[\boldsymbol{A}\left(\theta^{S}\right) \tau] \boldsymbol{P}^{*}(\boldsymbol{X}).  $$

We calculated ***P***(***X***,*τ*) after induction using the same MATLAB routines from the finite state projection method [24], or the equivalent functions in the Armadillo C++ library. We used finite state projection to verify that our estimated probability distributions were below the error threshold (*ε*≤10^−12^) for finite *M*; see below.

### Likelihood of smFISH data from probability distributions

The smFISH data are for a finite sample of cells at several time points {0,*τ*_1_,*τ*_2_,…,*τ*_*S*_} after induction. Each cell was in a state, i.e., number of active TSs and mRNA molecules, contained within [***x***_***1***_,***x***_***2***_,…,***x***_***N***_]^*T*^. The size (*N*) of the vector and matrix is determined by *N*=*p*(*M*+1), where *p* is the number of distinct promoter states per cell (*p*=3 for a two-state model and two alleles per cell, i.e., a cell can have zero, one, or two active TSs). *M* is the maximum number of mRNA molecules a cell can display, which could, in principle, be infinite. For practical purposes, we chose *M*=500 because it is finite and larger than the expected mRNA levels in our smFISH data. The smFISH data vector ***Y*** ^***t***^ for sample *t* is a count of observed cell states, where [*n*_1_,*n*_2_,…,*n*_*N*_]^*T*^. The likelihood of having sampled the observed data given the calculated distributions ***P***(***X***,*τ*) for model parameters *θ* is a product of multinomial distributions:

7 $$ \mathcal{L} = P(Y | \theta) = \prod_{t=0}^{S} \left[ \left(\frac{\left({\sum\nolimits}_{j} Y^{t}_{j}\right)!}{\prod_{k} Y^{t}_{k}!} \right) \prod_{i=1}^{N} [P(\boldsymbol{x_{i}},\tau_{t})]^{Y^{t}_{i}} \right].   $$

### Calculating the Bayesian posterior probability

The Bayesian posterior probability is the likelihood $\mathcal {L}$ multiplied by *P*(*θ*) and divided by *P*(*Y*), which are the prior probability distributions of the parameters and data. These priors are often unknown and *P*(*θ*) and *P*(*Y*) are presumed flat and constant, i.e., any parameter set and data set are equally likely. BayFish assumes flat priors unless specified otherwise. We implemented a Heaviside step function for *P*(*θ*), where the prior was zero for non-physiological parameters, but otherwise flat and constant. Non-physiological parameters include negative numbers (i.e., below 10^−8^) or a maximum transcription rate (i.e., 12–18 mRNAs per minute; see [31]).

### Validating BayFish with synthetic smFISH data

**Fig. 3:**
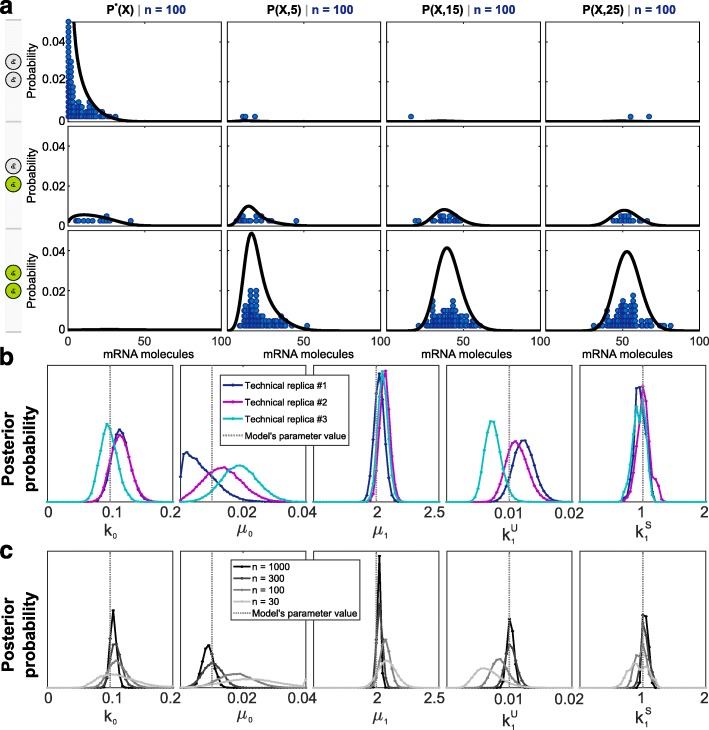
Validating BayFish on synthetic smFISH data. **a** Example of a technical replicate of synthetic smFISH data generated for an underlying *k*_1_-stimulus model, where *n*=100 cells per time point were sampled from the mRNA and active transcription site distribution (solid lines). Our synthetic data were sampled at *t*=0,5,15,25 min after induction. **b** Marginal Bayesian posterior distributions of parameters estimated by BayFish on three technical replicates (different colors). **c** Marginal Bayesian posterior distributions of parameters estimated by BayFish for different sampling densities (*n*=30,100,300,1000 cells per time point) of the same technical replicate. Vertical dashed lines are the true parameters of the *k*_1_-stimulus model used to generate the synthetic data

To test the ability of BayFish to infer parameters correctly, we generated synthetic smFISH data from a two-state model with known parameters. Our first model was a *k*_1_-stimulus model, where *k*_1_ changed from $k_{1}^{U}$ to $k_{1}^{S}$ upon induction and all other parameters stayed constant; see “Methods”. We created three technical replicates of synthetic smFISH data with a similar sampling density and number of time points as our *Npas4* data. Each technical replicate (Fig. 3 a) is different from the others only because of sampling error. We then ran BayFish using an underlying *k*_1_-stimulus model to infer the free parameters of each technical replicate. The mRNA degradation rate was not a free parameter in these BayFish runs and was fixed to its known value to mimic our situation for *Npas4*.

If the synthetic smFISH data were too sparse to constrain the model, then we would expect the Bayesian posterior distributions to be flat. However, each BayFish run converged to well-defined Bayesian posterior distributions of model parameters and the technical replicates had posterior distributions that were relatively close to one another and overlapped the true underlying parameters (Fig. 3 b). This demonstrates that sparsely sampled smFISH data at multiple time points already constrain the parameters of the underlying model. We then created a synthetic smFISH data set using the same *k*_1_-stimulus model, but varied the sampling density at each time point (*n*=30,100,300,1000 cells). As expected, increasing the sampling density better constrained the Bayesian posterior distribution and more accurately estimated the underlying model parameters (Fig. 3 c).

### Model selection using BayFish and information criteria

Previously, we initialized BayFish with the correct underlying model (*k*_1_-stimulus model). However, one does not usually know the correct model and it has to be inferred along with the unknown parameters. It is well known that models with more parameters have a higher likelihood of fitting the data. Thus, we combined BayFish with several likelihood-based metrics to evaluate different underlying models and penalize those with more free parameters (see “Methods”). These metrics are the Bayesian information criterion (BIC) [32] and the Akaike information criterion (AIC) [33], which are based on the maximum likelihood calculated by BayFish. The deviance information criterion (DIC) [34] uses both the likelihood and the Bayesian posterior distribution calculated by BayFish.

**Fig. 4:**
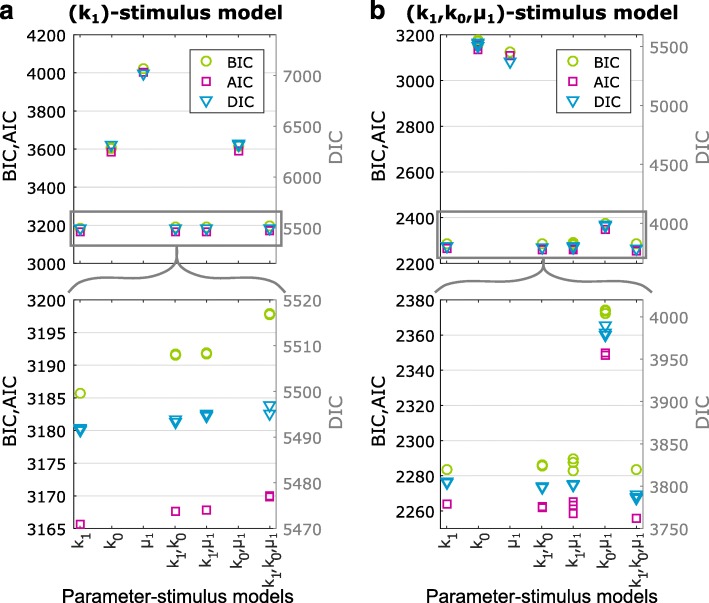
Model selection using BayFish and information criteria. We applied the Bayesian information criterion (BIC), the Akaike information criterion (AIC), and the deviance information criterion (DIC) metrics to the BayFish results obtained with the different parameter-stimulus models listed on the *x*-axis. All models were run on the same synthetic smFISH data. The maximum likelihood observed in each BayFish run was used for BIC and AIC metrics, and the full likelihood and Bayesian posterior distribution, excluding the burn-in period, were used for DIC. Models with the lowest BIC and AIC scores (left, *y*-axis) and DIC (right, *y*-axis) are the most informative models with the fewest parameters. **a** BayFish results for synthetic smFISH data (*n*=100 cells per time point) generated for an underlying *k*_1_-stimulus model. **b** BayFish results for synthetic smFISH data (*n*=100 cells per time point) generated for an underlying (*k*_1_,*k*_0_,*μ*_1_)-stimulus model. AIC Akaike information criterion, BIC Bayesian information criterion, DIC Deviance information criterion

To test the ability of BayFish and information criteria to select the correct model, we generated two synthetic smFISH data sets from different parameter-stimulus models. The first set was generated using a *k*_1_-stimulus model, whereas the second set was generated using a more complex (*k*_1_,*k*_0_,*μ*_1_)-stimulus model; see “Methods.” We then systematically ran BayFish using multiple underlying parameter-stimulus models, where different combinations of parameters were affected by the stimulus: *k*_1_-, *k*_0_-, *μ*_1_-, (*k*_1_,*μ*_1_)-, (*k*_0_,*μ*_1_)-, and (*k*_1_,*k*_0_,*μ*_1_)-stimulus models. The one-parameter-stimulus models had five free parameters and the three-parameter-stimulus model had seven free parameters to be inferred. As before, the mRNA degradation rate was fixed to its known value. We ran three replicas of BayFish with random initial parameters for *T*=10^5^ iterations for each underlying parameter-stimulus model. We then plotted the different information metrics obtained from each BayFish run on the *k*_1_-stimulus synthetic data set (Fig. 4 a) and the (*k*_1_, *k*_0_, *μ*_1_)-stimulus synthetic data set (Fig. 4 b). A lower information criterion score indicates that the underlying model had a better fit. Our results with synthetic data demonstrate that BayFish and the different information criteria select the correct underlying model.

### Running BayFish on *Npas4* smFISH data

**Fig. 5:**
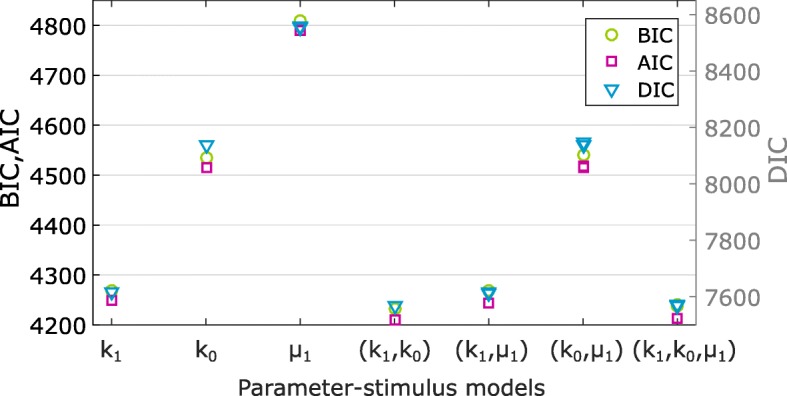
Comparing different stimulus models for *Npas4* smFISH data. We applied the BIC, AIC, and DIC metrics to the *Npas4* BayFish results obtained with the different parameter-stimulus models listed on the *x*-axis. For each parameter-stimulus model, three replicas of BayFish were run with different initial conditions. The Bayesian posterior distributions for each parameter-stimulus model are shown in Additional file 1: Figure S1. AIC Akaike information criterion, BIC Bayesian information criterion, DIC Deviance information criterion

**Fig. 6:**
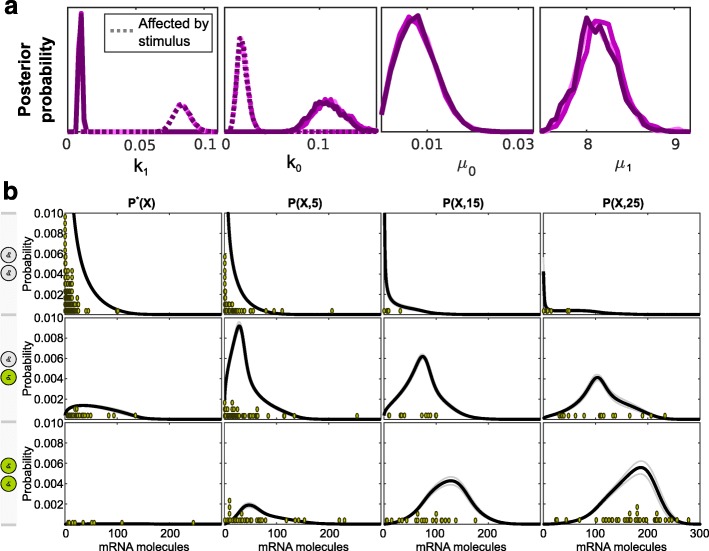
Bayesian posterior distribution for (*k*_1_,*k*_0_)-stimulus model run on *Npas4* smFISH data. **a** Marginal posterior distributions of parameters for BayFish replicas (i.e., different colors correspond to distinct random number generator seeds and initial conditions). There are two distributions for *k*_0_ and *k*_1_, one of which is the pre-stimulus parameter (continuous lines) and the other is the post-stimulus parameter (dotted lines). **b** The mean distribution of mRNA and active transcription site 〈***P***(***X***,*τ*)〉 as inferred from the Bayesian posterior distribution of parameters. The standard deviation (*σ*_**P**_) is shown in gray. A histogram of experimental data is shown for comparison (green dots)

We then used BayFish to infer parameters and select an underlying parameter-stimulus model for the *Npas4* smFISH data. We used the same approach as above, but the *Npas4* mRNA degradation rate constant was fixed to *δ*=0.0559 min ^−1^ [35]. Our results demonstrate that the best underlying model with the fewest parameters is the (*k*_1_,*k*_0_)-stimulus model (Fig. 5). The inferred mRNA and active TS distribution and Bayesian posterior distribution of the (*k*_1_,*k*_0_)-stimulus model are shown in Fig. 6 and summarized in Table 1. Model selection using BayFish and information criteria also showed that not all parameters are equivalent. Regulation of *k*_1_ by the stimulus consistently gave a better fit to the observed data than regulation by *k*_0_ or *μ*_1_ alone or in combination.

**Table 1:** Estimated parameters for *Npas4* (*k*_1_,*k*_0_)-stimulus model

	Parameter	Mean	Standard deviation	Units
$k_{1}^{U}$	Activation rate	0.0093	0.0010	*min* ^−1^
$k_{1}^{S}$	Activation rate after stimulus	0.0839	0.0063	min ^−1^
$k_{0}^{U}$	Deactivation rate	0.1108	0.0172	min ^−1^
$k_{0}^{S}$	Deactivation rate after stimulus	0.0189	0.0056	min ^−1^
*μ*_0_	*ρ*_0_ synthesis rate	0.0078	0.0042	mRNA min ^−1^
*μ*_1_	*ρ*_1_ synthesis rate	8.14	0.2305	mRNA min ^−1^
*δ*	mRNA degradation rate	0.0559	–	min ^−1^

## Discussion

Like any inference approach, BayFish is limited by the information content of the data and the underlying model assumptions. For example, our smFISH data measured the mRNA and active TS counts per cell, but one could also measure the brightness of TS sites from the smFISH data to estimate the number of nascent mRNAs and, hence, the transcription rate (*μ*_1_) [20]. This additional information could further constrain the underlying model, as has been done by others [15]. We did not include or fit TS intensity in our *Npas4* model and, thus, this provides an independent test of the *μ*_1_ parameter inferred by BayFish. We estimated nascent mRNAs and the transcription rate from *Npas4* active TSs (Additional file 1: Figure S2). The estimated transcription rate has a strong mode between 7–10 mRNA min ^−1^, which is consistent with our inferred *μ*_1_ of 8 mRNA min ^−1^; see Table 1.

However, our analysis also shows that there are caveats with estimating the transcription rate from the integrated intensity of active TSs. First, the estimate depends on the choice of transcription elongation rate, which can vary across genes and organisms [31]. Second, some active TSs have more nascent mRNAs and a higher transcription rate than theoretically possible (grey area in Additional file 1: Figure S2). The simplest explanation for these unusually bright spots is that mRNAs continue to be associated with chromatin at active TSs after transcription until further processing [36]. Thus, the integrated intensity of an active TS cannot be assumed to represent only nascent mRNAs in the process of transcription.

Our mathematical model of stochastic gene expression also assumed that each promoter allele was regulated independently [11, 15, 23, 27]. However, previous work has also shown that genes can exhibit strongly correlated gene expression, particularly when integrated adjacent to one another on the same chromosome [14, 37]. If one *Npas4* allele is independent of the other, then we expect the active TS to exhibit a binomial distribution of zero, one, or two active TSs with probability (1−*p*)^2^, 2*p*(1−*p*), or *p* ^2^ where *p*=*k*_1_/(*k*_0_+*k*_1_), i.e., the probability of an active allele or burst fraction. Although our results show a statistically significant difference between the measured and expected fractions for independent alleles (Additional file 1: Figure S3), the data are closer to independent alleles than perfectly correlated alleles (i.e., there are no cells with one active TS). Modeling the weak correlations between alleles and the post-transcriptional processing of mRNAs at active TSs is beyond the scope of our current software package, but these could be potentially informative extensions of BayFish.

## Conclusions

We developed a suite of MATLAB programs (BayFish), and an alternative C++ version, that use Bayesian inference to estimate model parameters robustly from smFISH data. We expect this software package to be useful for other labs because it fills a critical gap in the downstream analysis of population snapshots of smFISH in single cells. The user specifies any mathematical model of stochastic gene expression with an unknown set of parameters (*θ*) and provides smFISH data (*Y*) of mRNA and active TS counts in a population of cells at different time points before and after induction. BayFish uses a Monte Carlo method to estimate the Bayesian posterior probability *P*(*θ*|*Y*) of the model parameters, which elucidates the best-fitting parameters and quantifies their uncertainty. Based on the confidence intervals of inferred parameters from a current data set, BayFish permits labs to design the next set of experiments and collect additional smFISH data (e.g., different times or more cells) that is maximally informative.

We generated synthetic data to validate the ability of BayFish to infer the correct parameters and tested its performance on smFISH data sets with sampling error. We further demonstrated how BayFish can be combined with information criteria to select the most informative underlying model. Finally, we used BayFish to extract meaningful biological information from *Npas4* gene expression in single neurons (Fig. 1). Our results favor a two-state model where the stimulus increases *k*_1_ and decreases *k*_0_. Both parameters modulate the *Npas4* burst fraction, e.g., fraction of time that a promoter spends in the active, on state, without changing the transcription rate of the on or off state. Modulation of the burst fraction upon induction is consistent with previous observations for other genes [13, 15, 38], although modulation of the transcription rate (*μ*_1_) upon induction has also been documented [14]. Future experiments will address mechanisms of activation and cell-to-cell variability in *Npas4* and other immediate-early genes of primary neurons. This can be done by combining genetic and pharmacological perturbations of gene expression with downstream BayFish analysis of multi-color smFISH distributions of several immediate-early genes.

## Methods

### *Npas4* smFISH measurements in single neurons

Neuron-enriched cultures were generated from the cortex of male and female E16.5 CD1 mouse embryos (Charles River Laboratories Inc., Wilmington, MA, USA) and cultured as previously described [39]. Neurons were treated with 1 µM tetrodotoxin (TTX) (Tocris Cookson, Ballwin, MO, USA), a sodium channel inhibitor, at DIV6 and depolarized by elevating the extracellular potassium concentration to 55 mM with an isotonic KCl solution at DIV7 [40], which activates L-type voltage-gated calcium channel dependent transcription of *Npas4* [41]. Cells were fixed at four time points: no KCl, 5 min KCl treatment, 5 min KCl treatment plus 10 min condition medium, and 5 min KCl treatment plus 20 min condition medium as indicated in Fig. 1.

Neurons were fixed in 4% Paraformaldehyde (PFA) at room temperature for 10 min after sampling and permeabilized by 70% (v/v) EtOH at 4 °C overnight. The mouse *Npas4* mRNAs were hybridized with the Quasar^®;^ 570 Stellaris RNA FISH Probe set following the manufacturer’s instructions, which are available online. Custom Stellaris^®;^ FISH Probes were designed against mouse *Npas4* mRNA by utilizing the Stellaris^®;^ RNA FISH Probe Designer (Biosearch Technologies, Inc., Petaluma, CA, USA), which is available online. We hybridized probes to samples in a hybridization buffer (10% formamide, 10% 20 × SSC, 10% dextran sulfate, 1 mg mL ^−1^ *Escherichia coli* tRNA, 2 mM vanadyl ribonucleoside complex, and 20 µg mL ^−1^ Bovine serum albumin (BSA)) at 37 °C for 4 hours followed by Hoechst staining. Z-stack images were captured on a wide-field microscope (DMI4000, Leica) equipped with a CCD camera (DFC365 FX, Leica) and controlled by MetaMorph (Molecular Devices). An objective with NA 1.4 and 63 × magnification yielded an *xy* pixel-size of 146 nm. Then, 35–45 Z-slices were recorded with a 200 nm step-size and 1 second exposure time.

We used FISH-quant [21] to identify and count absolute mRNA numbers and active TSs in single cells (Fig. 1). The active TSs can be detected because nascent mRNAs are transiently attached to the elongating RNA Polymerase II in the gene, accumulating fluorescent probes around active sites, and then appear as highly intense dots (one or two, as there are two copies of the gene) in the nucleus of the diploid cell. We and others have confirmed that these nuclear spots mark the active TSs because they colocalize in two-color smFISH with probes specific for the gene introns, which are present only in nascent RNAs (data not shown and [42]).

### Monte Carlo sampling and burn-in

The number of iterations (*T*), covariance matrix (*Σ*), and burn-in period were determined by monitoring the acceptance rate of proposals and the distribution of parameters and likelihood in the stationary phase of the Monte Carlo algorithm. The rate at which the Markov chain approaches stationarity (i.e., the region with higher likelihood) depends on the covariance matrix *Σ* used to draw new proposals. We defined the burn-in as the initial period where the log-likelihood was increasing and less than 99.5*%* of the maximum. The burn-in period is sensitive to the initial parameters and the parameter-stimulus model. Given our experimental data, we verified that *T*=10^5^ iterations and our covariance matrix *Σ* were sufficient for BayFish to achieve stationarity and adequately sample the Bayesian posterior distribution after discarding the burn-in. The final covariance matrix *Σ* was diagonal with 10^−5^ for *k*_0_,*k*_1_,*μ*_0_ and 10^−3^ for *μ*_1_ proposals.

### Generating synthetic smFISH data

For a given stimulus model and known parameter set (truth), we calculated the mRNA and active TS distributions using the algorithms described in the main text. The pre-stimulus stationary distribution at *t*=0 min was generated using the unstimulated parameters, whereas the post-stimulus distributions at *t*=5,15,25 min were generated using the stimulated parameters. From these distributions, we created a technical replicate by randomly sampling *n* cells from each mRNA and active TS distribution calculated at each time point. We generated technical replicates of synthetic smFISH data for two parameter-stimulus models: a *k*_1_-stimulus and a (*k*_1_, *k*_0_, *μ*_1_)-stimulus model. The *k*_1_-stimulus model had the following six parameters: $k_{1}^{U} = 0.01$ min ^−1^, $k_{1}^{S} = 1$ min ^−1^, *k*_0_=0.1 min ^−1^, *μ*_1_=2 mRNA min ^−1^, *μ*_0_=0.01 mRNA min ^−1^, and *δ*=0.05 min ^−1^. The (*k*_1_, *k*_0_, *μ*_1_)-stimulus model had the following eight parameters: $k_{1}^{U} = 0.01$ min ^−1^, $k_{1}^{S} = 1$ min ^−1^, $k_{0}^{U} = 1$ min ^−1^, $k_{0}^{S} = 0.01$ min ^−1^, $\mu _{1}^{U} = 0.2$ mRNA min ^−1^, $\mu _{1}^{S} = 2$ mRNA min ^−1^, *μ*_0_=0.01 mRNA min ^−1^, and *δ*=0.05 min ^−1^.

### Information criterion and model fitting

We used several information criteria, such as BIC [32], AIC [33], and DIC [34], to evaluate the likelihood of different models and to penalize model over-fitting:

- Bayesian information criterion:
  8 $$ \text{BIC} = -2 \ln(\hat{\mathcal{L}}) + m \ln (n).  $$
- Akaike information criterion:
  9 $$ \text{AIC} = -2 \ln (\hat{\mathcal{L}}) - 2 m + \frac{2m(m+1)}{n-m-1}.  $$

The maximum likelihood $\hat {\mathcal {L}}= P(Y|\hat {\theta })$ is the maximum value of $\mathcal {L}$ obtained during the BayFish run, *m* is the number of free parameters that were fit, and *n* is the total sample size. These metrics do not take full advantage of the Bayesian posterior probability estimated by BayFish. Thus, we also used:

- Deviance information criterion:
  10 $$ \text{DIC} = 2 \bar{D} - D(\bar{\theta}).  $$

The deviance is

11 $$ D(\theta) = -2 \ln P(Y|\theta) = -2 \ln \mathcal{L}.  $$

$\bar {D} = E[D(\theta)]$ is the mean of the deviance *D*(*θ*) calculated from the Bayesian posterior probability, whereas $D(\bar {\theta }) = D(E[\theta ])$ is the deviance of the mean of *θ* calculated from the Bayesian posterior probability.

## Availability and requirements

**Project name:** BayFish

**Project homepage:** https://github.com/mgschiavon/BayFish; http://doi.org/10.5281/zenodo.830056

**Operating system:** Platform independent

**Programming language:** MATLAB or C++

**Other requirements:** See README file in the project homepage.

**License:** GNU General Public License v3.0

Additionally, the datasets analyzed during the current study are also available in the GitHub repository, https://github.com/mgschiavon/BayFish/tree/master/DATA.

## Additional file

Additional file 1 Supplementary figures S1–S3. (PDF 233 kb)

## Abbreviations

AIC: Akaike information criterion
BIC: Bayesian information criterion
CME: Chemical master equation
DIC: Deviance information criterion
FISH: Fluorescence in situ hybridization
RNA: Ribonucleic acid
smFISH: Single-molecule RNA FISH
TS: transcription site
