# Supplementary material for: BayFish: Bayesian inference of transcription dynamics from population snapshots of single-molecule RNA FISH in single cells
Source: Genome Biol. 2017 Sep 4;18:164. doi: 10.1186/s13059-017-1297-9 (PMC5582403; doi:10.1186/s13059-017-1297-9)
Supplement: Additional file 1 — Supplementary figures S1–S3. (PDF 233 kb) [file 13059_2017_1297_MOESM1_ESM.pdf]

BayFish: Bayesian inference of transcription dynamics from population snapshots of single-molecule RNA FISH in single cells

Mariana Gómez-Schiavon, Liang-Fu Chen, Anne E. West, and Nicolas E. Buchler

August, 2017

Abstract

Single-molecule RNA fluorescence in situ hybridization (smFISH) provides unparalleled resolution in the measurement of the abundance and localization of nascent and mature RNA transcripts in fixed, single cells. We developed a computational pipeline (BayFish) to infer kinetic parameters of gene expression from smFISH data at multiple time points after gene induction. Given an underlying model of gene expression, BayFish uses a Monte Carlo method to estimate the Bayesian posterior probability of the model parameters and quantify the parameter uncertainty given the observed smFISH data. We tested BayFish on synthetic data and smFISH measurements of the neuronal activity inducible gene Npas4 in primary neurons.

References

[1] Mueller F, Senecal A, Tantale K, Marie-Nelly H, Ly N, Collin O, et al. FISH-quant: automatic counting of transcripts in 3D FISH images. Nature Methods. 2013 mar;10(4):277–278.

[2] Bahar Halpern K, Itzkovitz S. Single molecule approaches for quantifying transcription and degradation rates in intact mammalian tissues. Methods. 2016 apr;98:134–142.

[3] Struhl K. Transcriptional noise and the fidelity of initiation by RNA polymerase II. Nature Structural & Molecular Biology. 2007 feb;14(2):103–105.

Supplementary figures

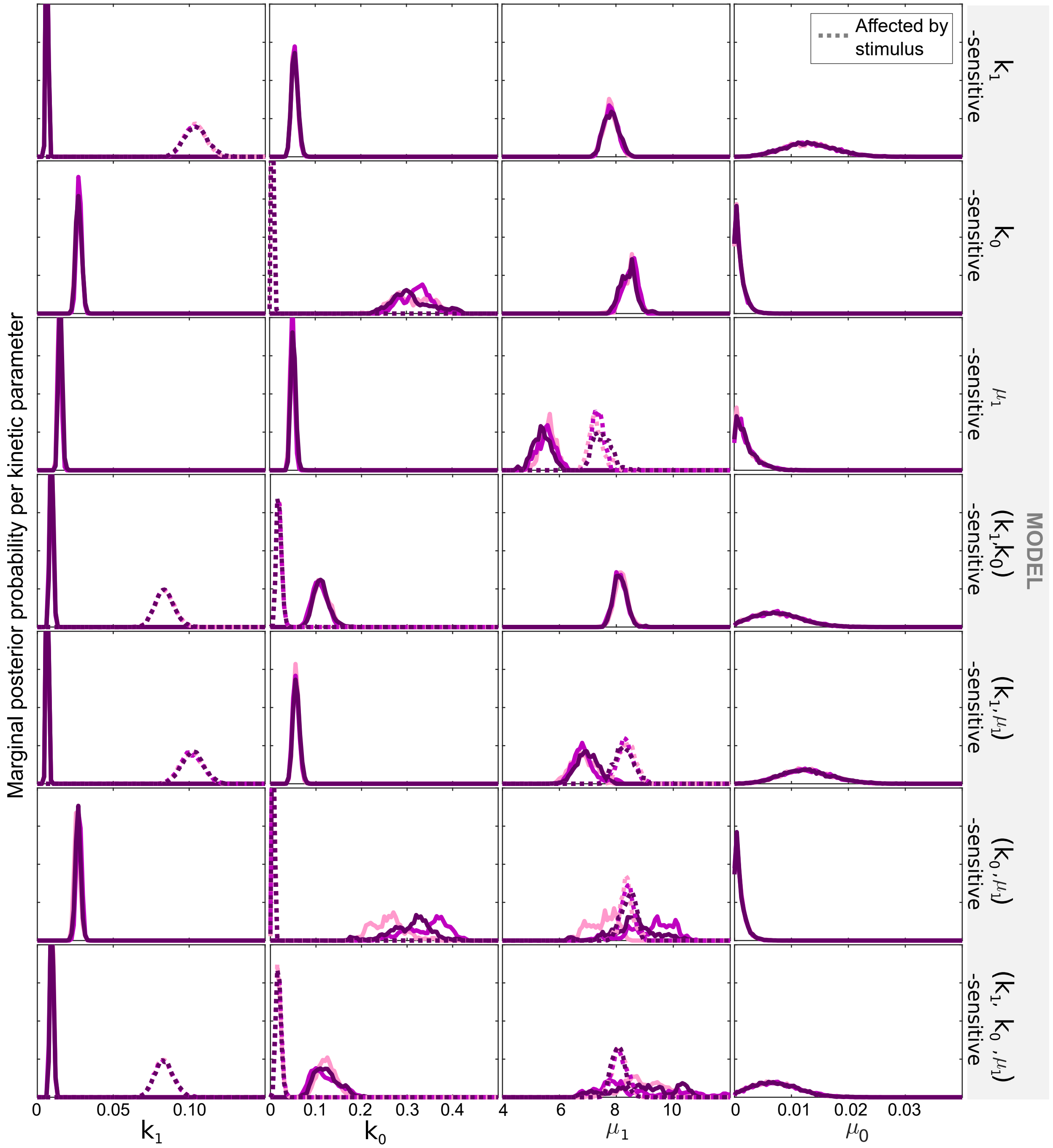

[!h]

Figure S1: **Bayesian posterior distributions for different parameter-stimulus models run on Npas4 smFISH data.** Each row displays the marginal posterior distributions of three BayFish runs initialized with different parameters for the different parameter-stimulus models indicated to the left. Each column plots the marginal posterior distribution of the same model parameter(s). For those parameters that are sensitive to stimulus, we plot the uninduced posterior distributions as solid lines and the post-stimulus posterior distributions as dotted lines.

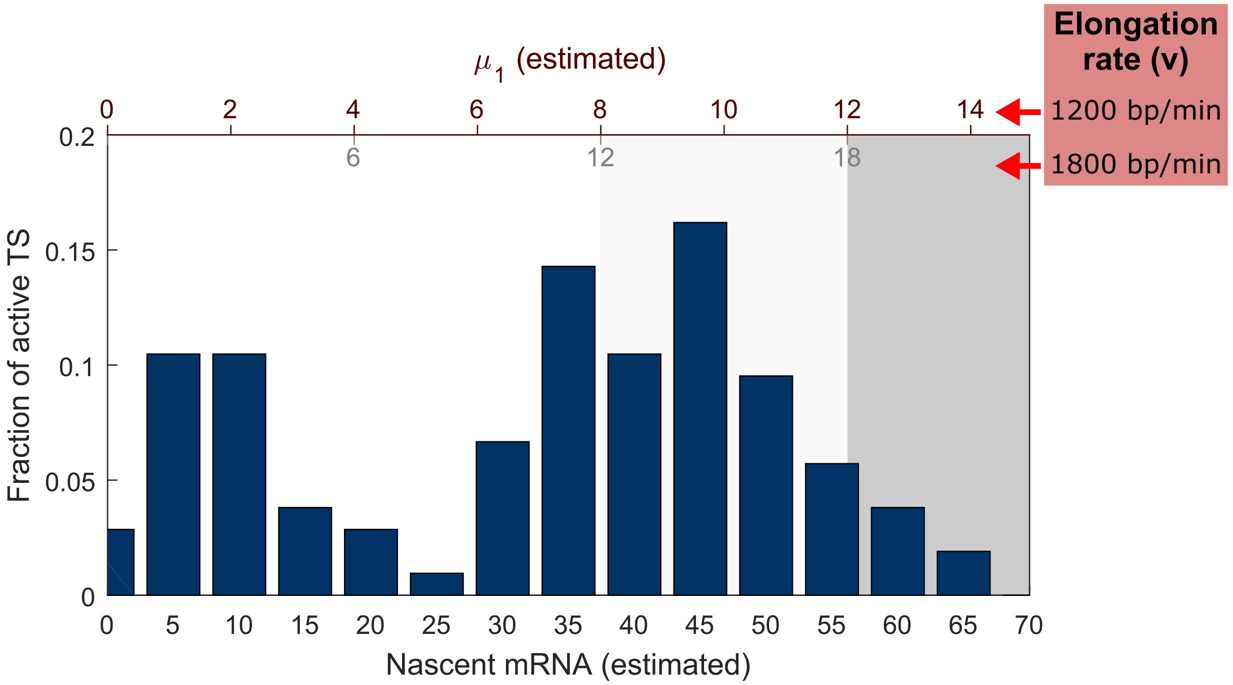

Figure S2: **Estimate of Npas4 nascent mRNAs and transcription rate in active TS.** We measured the integrated intensity of each active TS at 25 minutes after induction using FISH-quant [1]. The integrated intensity was converted into nascent mRNAs and transcription rate (μ_1) using the formulas described in [2]. We used a Npas4 probe coverage weight factor of $W = 0.571$ and a polymerase elongation rate of 1200-1800 bp per minute [3]. Shaded area corresponds to transcription rates beyond the maximum limit.

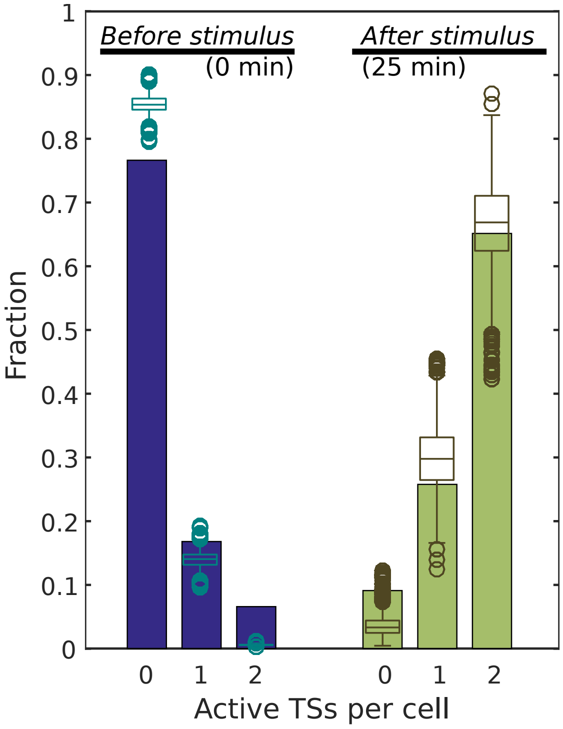

Figure S3: **The distribution of active TSs suggests that Npas4 alleles are mostly independent.** The colored bars are the measured fraction of cells with zero, one or two active TSs before stimulus ($t = 0$ minutes; $n = 137$ cells) and after stimulus ($t = 25$ minutes after induction; $n = 66$ cells). These timepoints are closest to a time-independent, stationary distribution where the fraction of active TS is expected to follow a binomial distribution. The boxplots show the expected fraction of cells with zero, one or two active TSs given the Bayesian posterior distribution of k_1,k_0 before and after the stimulus (Figure 6). The central line indicates the median, the bottom and top edges of the box are the 25th and 75th percentiles; whiskers extend to the most extreme data points not considered outliers, and outliers are plotted as circles.
